# SARS-CoV-2 and COVID-19: revisiting the most important research questions

**DOI:** 10.1186/s13578-021-00730-1

**Published:** 2021-12-18

**Authors:** Kit-San Yuen, Zi-Wei Ye, Sin-Yee Fung, Pak-Hin Hinson Cheung, Chi-Ping Chan, Dong-Yan Jin

**Affiliations:** 1 School of Biomedical Sciences, The University of Hong Kong, 3/F Laboratory Block, 21 Sassoon Road, Pokfulam, Hong Kong; 2 School of Medical and Health Sciences, Tung Wah College, Kowloon, Hong Kong; 3 Department of Microbiology, The University of Hong Kong, Pokfulam, Hong Kong

**Keywords:** SARS-CoV-2, COVID-19, Pandemic, Endemic, Vaccines, Antivirals

## Abstract

In February 2020, we highlighted the top nine important research questions on SARS-CoV-2 and COVID-19 concerning virus transmission, asymptomatic and presymptomatic virus shedding, diagnosis, treatment, vaccine development, origin of virus and viral pathogenesis. These and related questions are revisited at the end of 2021 to shed light on the roadmap of bringing an end to the pandemic.

When only slightly more than 80,000 confirmed cases of SARS-CoV-2 infection were reported worldwide and right before COVID-19 was declared a pandemic by the WHO, we discussed the nine most important research questions on SARS-CoV-2 and COVID-19, in our commentary that was published in *Cell & Bioscience* at the end of February 2020 [1]. These questions are:

1. How was SARS-CoV-2 transmitted in Wuhan?
2. How transmissible and pathogenic is SARS-CoV-2 after passages in humans?
3. How important is asymptomatic and presymptomatic virus shedding in SARS-CoV-2 transmission?
4. Is fecal-oral route important in SARS-CoV-2 transmission?
5. How should COVID-19 be diagnosed and what diagnostic reagents should be made available?
6. How should COVID-19 be treated and what treatment options should be made available?
7. Are inactivated vaccines a viable option for SARS-CoV-2?
8. What were the origins of SARS-CoV-2 and COVID-19?
9. Why is SARS-CoV-2 less pathogenic compared to SARS-CoV?

Science has contributed substantially to the battle against COVID-19 in the past two years. Particularly, prophylactic vaccines and therapeutic drugs have been made available quickly, which have brought about and will continue to bring about a big difference in prevention and control of COVID-19. At the end of 2021, we have much better answers to all nine questions listed above and asked at the beginning of 2020. The recent efforts in our research community are also reflected in the Special Issue in *Cell & Bioscience* dedicated to “Frontiers in Virology and COVID-19”. As a preface to the Special Issue, we will revisit the nine research questions and other related issues. This will not only give an account of how far we have gone since early 2020, but also provide a personal perspective of what is needed to end the pandemic.

Concerning the first question about SARS-CoV-2 transmission pattern in Wuhan, results from population-level testing of antibodies against SARS-CoV-2 conducted in mid-2020 in residents of Wuhan [2] have confirmed several of our initial predictions. First, since 82.1% of infected individuals were asymptomatic as confirmed by the study [2], the numbers of undiagnosed infected people and asymptomatic carriers would be much higher than the original estimates. Second, the rise in the number of confirmed cases in Wuhan after lockdown was plausibly ascribed to multiple reasons including high basal number of infected people before the lockdown and high attack rate within families. Third, the decision to lock down Wuhan and nearby cities was extraordinary, but lifesaving. It was based on key scientific evidence obtained in January 2020 from Shenzhen [3] and the visionary prediction that the scale of the outbreak was already much bigger than what was recognized. Reconstruction of the transmission chains in 1178 patients with COVID-19 diagnosed in early 2020 in Hunan is most revealing about the role of superspreading in the transmissions of SARS-CoV-2 [4]. Hunan is a Chinese province next to Hubei, to which the city of Wuhan belongs. Importantly, 80% of the secondary infections were caused by only 15% of the infected individuals, whereas most of the remaining 85% did not transmit the virus to anyone else. About 80% of the droplets and aerosol particles that contain SARS-CoV-2 were emitted by 20% of infected people. More droplets and aerosols are generated by people who are older, obese or immunocompromised. Subjects who have higher lung capacities, speak louder, are physically more active or are singing also emit more virus than their counterparts. From another perspective, about 90% of the SARS-CoV-2 virus circulating in the communities at any given time was carried by only 2% of infected people, who have the potential to become superspreaders. At least 50% of the remaining individuals tested positive for SARS-CoV-2 RNA are non-contagious. Consistently, a small subset of people account for the biggest share of transmissions. It is particularly noteworthy that superspreading commonly occurs unexpectedly in people who have mild or no symptoms. The pandemic is fueled by active transmission in young, healthy and mobile groups of people. Based on the understanding that about 10% of the locations account for 80% of the transmissions, control measures that target transmission hotspots such as restaurants, cafes, gyms, karaoke spots and party rooms are most effective in curtailing the pandemic, when compared to a complete lockdown. Obviously, these hotspots vary in different places. For example, superspreading events have also occurred in different settings such as construction sites, labor-intensive firms, food production facilities, schools, prisons, religious gatherings, multilevel marketing activities, dancing classes and dormitories, where many people are crowded into confined and poorly ventilated spaces. In this regard, the Japanese approach of “avoiding the three Cs (closed spaces, crowded places and close-contact settings)” has also been successful in preventing explosive outbreaks. Hence, it will be much more effective if mitigations are specifically designed and executed to target superspreading rather than to achieve and maintain a SARS-CoV-2-free status. For example, identification and quarantine of people with high viral loads are much more cost-effective and efficient in the prevention of superspreading and explosive outbreaks, if compared to blocking all visitors from outside of the country or quarantining them blindly for 28 days.

We have most answers to the second question concerning transmissibility and pathogenicity of SARS-CoV-2 after passages in humans. Highly transmissible variants of SARS-CoV-2 such as D614G and Alpha variants have quickly replaced the original Wuhan strain worldwide, but they gave way to the highly fit and even more transmissible Delta variant, which accounts for 99% of sequenced COVID-19 cases globally at present. It remains to be seen whether the Omicron variant newly identified from South Africa and carrying more than 50 point mutations in the genome, among which more than 30 are in the spike region, might outcompete Delta to become the next predominant strain in the near future. Noteworthily, the pathogenicity of most if not all highly transmissible strains does not increase. More and more people have developed immunity against SARS-CoV-2 through vaccination and/or natural infection. Although breakthrough infections can still occur when neutralizing antibodies wane or are intrinsically insufficient due to different reasons, in most cases severity of disease decreases substantially. Thus, COVID-19 will be more and more like common cold caused by community-acquired human coronaviruses. In support of this notion, an analysis of 569,742 cases of COVID-19 collected from multiple sites in US indicated a death rate of 0.1% among vaccinated people infected with Delta variant of COVID-19 [5], which is much lower than those of 1.3% or 1.6% among unvaccinated people and comparable to that of seasonal influenza. Thus, even if the pathogenicity of Delta variant remains unchanged compared to that of the original strain, it will cause less harm where rates of vaccination and/or previous natural infection are high. This is very similar to the 1918 pandemic strain of influenza. Although it is highly pathogenic, the 1918 pandemic virus will not cause another pandemic now since existing immunity against it is strong and widespread among healthy people of different age groups.

The importance of asymptomatic and presymptomatic virus shedding in SARS-CoV-2 transmission, which was the third question asked in early 2020, has now been fully established. Asymptomatic and presymptomatic virus shedding poses a great challenge to infection control [3]. A small subset of asymptomatic and presymptomatic people infected with SARS-CoV-2 shed a large amount of virus and are potential superspreaders. It is also noteworthy that SARS-CoV-2 establishes a persistent infection and undergoes rapid evolution in severely immunocompromised people, giving rise to mutations that foreshadow the circulating variants [6]. Indeed, many variants of concern and variants of interest, including the newly identified Omicron variant, are thought to be arisen from immunocompromised subjects. Thus, immunocompromised individuals persistently infected with SARS-CoV-2 should be closely monitored. Uninfected but immunocompromised people should be given the priority to receive booster vaccination as many times as required. Persistent infection in some immunocompromised people might account for a small subset of cases suffering from long COVID-19. The term has no strict definition and usually refers to conditions when new and ongoing symptoms of COVID-19 are seen four or more weeks after the first onset. Some people either having long COVID-19 or being asymptomatic could be tested positive for SARS-CoV-2 RNA long after the first infection. However, if the viral titer is low, the viral RNA detected could be derived from viral gene fragments or non-functional pieces. These people with low viral titers are therefore considered non-contagious or minimally contagious.

Concerning the fourth question, we have a much better understanding of the transmission routes of SARS-CoV-2 now compared to two years ago. CDC categorizes modes of SARS-CoV-2 transmission as inhalation of droplets and aerosol particles that contain virus; deposition of droplets and aerosols on exposed mucous membranes in the nose, mouth and eyes; and touching the mucous membranes with soiled hands contaminated with virus either directly via droplets and aerosols or indirectly by touching surfaces. The definitions of droplets of more than 100 μm in diameter and aerosols of less than 100 μm have been updated. Whereas droplets are liquid particles that settle out of the air within seconds and minutes, aerosols are stable suspension of solid and/or liquid particles dried from fine droplets and capable of staying from minutes to hours. Airborne transmission has been verified as one major route for the spread of SARS-CoV-2. Particularly, as mentioned above, enclosed spaces with inadequate ventilation or air conditioning are high-risk spots for superspreading. In contrast, transmission of SARS-CoV-2 via contaminated surfaces, including packaging materials, frozen or chilled seafoods and meats, is not known to contribute substantially to new infections. In this regard, the hypothesis that several outbreaks in China and even the original one connected to the Huanan Seafood Wholesale Market in Wuhan were caused by imported foods or materials contaminated with SARS-CoV-2, has to be supported by more direct and concrete evidence. Gastrointestinal symptoms such as diarrhea, vomiting and abdominal pain are not uncommon but can be seen in up to 20% of all patients. Prolonged shedding of SARS-CoV-2 from gastrointestinal tract for as long as a month has been observed in a subset of adult and pediatric patients who are either symptomatic or asymptomatic. Interestingly, gastrointestinal involvement appears to alleviate the severity and reduce the mortality of disease to some extent. Although detection of viral RNA in sewage has been used successfully to trace infected individuals, prolonged virus shedding in the feces of convalescent and other lowly contagious patients raises a concern about its role in the prevention of superspreading. Circumstantial evidence in support of fecal-oral transmission of SARS-CoV-2 has been found in different settings such as transmission chains in high-rise buildings. However, this does not change the three main routes of transmission summarized above or the prevention methods including physical distancing, use of surgical masks, adequate ventilation, good hand hygiene and avoidance of 3Cs. SARS-CoV-2 in the feces might be transmitted via inhalation, disposition on the mucous membrane or touching the mucous membrane with contaminated hands. For the first two modes of transmission, virus in the feces has to be aerosolized.

As far as question five is concerned, all desired diagnostic reagents for SARS-CoV-2 infection have now been made available. Whereas quantitative RT-PCR analysis of SARS-CoV-2 RNA remains the gold standard in diagnosing SARS-CoV-2 infection, antigen and antibody tests also play critical roles in rapid screening, contact tracing, early detection of superspreading, assessment of immune protection and formulation of vaccination strategies. There is a need to sequence more SARS-CoV-2-positive samples for better identification and tracking of variants of concern and variants of interest, and for reconstitution of transmission chains. At present, this is particularly important in the monitoring of Omicron variant in Africa and elsewhere. Schemes and parameters for risk stratification are also required. For example, the lower the viral RNA titer, the lower the risk of transmission or superspreading. Based on this generally accepted concept, people with a very high Ct value in viral RNA detection (e.g. >30) and detectable neutralizing antibodies (e.g. measured by the surrogate test for antibodies directing against receptor-binding domain) might be exempted for quarantine.

For the sixth question concerning treatment options, two anti-SARS-CoV-2 agents have just been shown effective in clinical trials. In addition to remdesivir, corticosteroids and neutralizing antibodies that are already in clinical use, they promise to provide new treatment options to patients with COVID-19, particularly those who have higher risks of progressing to severe disease. The first oral anti-SARS-CoV-2 drug known as molnupiravir developed by Merck is a nucleoside analog that targets RNA-dependent RNA polymerase of SARS-CoV-2 to boost RNA mutagenesis [7]. It is just approved in November 2021 by UK’s regulatory agency for the treatment of mild-to-moderate COVID-19 in adults with a positive SARS-CoV-2 diagnostic test and who have at least one risk factor for developing severe disease. Prior to this, remdesivir and two monoclonal antibodies were first approved for clinical use in October 2020. The second oral anti-SARS-CoV-2 drug named poxlovid developed by Pfizer is a protease inhibitor that targets the main protease of SARS-CoV-2. It binds covalently to the catalytic cysteine residue of main protease. It is also known that the combination of poxlovid with ritonavir decelerates metabolism of poxlovid. Pfizer is currently seeking approval for emergency use of poxlovid from FDA. Molnupiravir and poxlovid are new weapons in the battle against COVID-19. They will be on the market in the coming weeks. However, they have to be used as early as practically possible after infection to have optimal beneficial effect. Since molnupiravir can evade or overcome the action of the proofreading enzyme of SARS-CoV-2, it might be more difficult for the virus to develop resistance [8]. However, it remains to be seen how quickly or how efficiently SARS-CoV-2 might develop resistance against molnupiravir or poxlovid after their widespread use.

Rapid development of effective vaccines against SARS-CoV-2 is heralded as one major scientific achievement in our battle to conquer COVID-19. We asked in our original question seven whether inactivated vaccines are a viable option for SARS-CoV-2. Indeed, they are a viable option, but mRNA vaccines, adenoviral vectored vaccines and subunit vaccines are not only feasible, but also superior as far as efficacy and duration of protection are concerned. Both mRNA vaccines and adenoviral vectored vaccines are the first-in-class approved for human use, so they truly represent major breakthroughs in molecular approaches to vaccine development. In contrast, although the inactivated vaccines can also elicit neutralizing antibodies, the titers of these antibodies are much lower compared to those in recipients of mRNA vaccines and in people recovered from moderate to severe COVID-19. As a result, the neutralizing antibodies wane to almost zero in almost all vaccinees within three months. Thus, similar to asymptomatic carriers with low viral titers, one or more booster vaccinations are required for recipients of inactivated vaccines. It remains to be determined whether additional booster injections have to be given on a regular basis. Plausibly, there should be much room for improvement in further refinement of the inactivated vaccines. In particular, the efficacy and duration of protection might be increased by elevating the dose of antigen, optimizing the use of adjuvants, optimizing the intervals between the first and second or between the second and third injections, as well as mixing and matching with other types of vaccines including mRNA, adenoviral vectored and subunit vaccines. The whole process through which an mRNA vaccine elicits humoral and cellular immunity resembles what happens when spike protein is expressed from the subgenomic RNA of SARS-CoV-2 during natural infection. In addition, the use of a very high dose of mRNA ensures that an overwhelming amount of spike protein is expressed and recognized by the host. Although mRNA vaccines are highly effective, they cannot stimulate mucosal and sterilizing immunity required for eradication of SARS-CoV-2 from humans. Thus, in addition to the development of variant-specific mRNA vaccines, particularly those targeting Delta and Omicron variants, which are already in the pipeline, live attenuated vaccines, replication-defective vaccines and single-cycle vaccines that can elicit mucosal immunity should continue to be explored. The widespread use of mRNA vaccines also provides an opportunity for booster immunization with these mucosal vaccines that are administered intranasally. This reminds us of the past success in the combination of Salk and Sabin vaccines in the near-eradication of poliovirus. Although cell-mediated immunity might still play a role in the alleviation of severe disease, neutralizing antibodies are the generally accepted correlates of immune protection against SARS-CoV-2 infection and symptomatic COVID-19. Estimates of neutralizing antibody levels required to prevention infection or severe COVID-19 are also available [9]. The tendency of the COVID-19 pandemic to become endemic just like the four community-acquired human coronaviruses, namely 229E, OC43, HKU1 and NL63 is evident. SARS-CoV-2 will unlikely be eradicated from humans in the near future. Eradication might not be necessary if the mortality is lower than 0.1%. However, eradication remains possible as long as our next-generation vaccines are sufficiently good. Effective vaccines against SARS-CoV-2 have been available, although some are more effective than the others. Increasing the production of the most effective vaccines, increasing vaccine accessibility in resource-limited countries, and educating the general public about the effectiveness and safety profiles of vaccines are three important tasks that hold the key to putting an end to the pandemic. There are new opportunities for international leaders and the WHO to show leadership and determination. Pooling vaccine manufacturing capabilities to make the most effective vaccines is in the best interest of all people on the globe. A mechanism to determine how vaccines should be made to target predominant variants, similar to the existing one by which WHO recommends the composition of the influenza vaccines for the next annual season, should also be considered and implemented. Vaccine hesitancy has become one major threat to our effort to curtail the pandemic. Scientists have a role to play here and have to work together with all stake holders to appeal to the different groups and to relieve their key concerns. Vaccination schemes have to be designed specifically for the immunocompromised and the adolescent groups to ensure high effectiveness and safety. The benefits that outweigh the risks should be explained more explicitly, transparently, and effectively to people in high-risk groups including those who are elderly, pregnant, immunocompromised, or suffering from chronic underlying diseases.

Our original response to the eighth question regarding the origins of SARS-CoV-2 and COVID-19 is still valid. Little progress has been made in the past 2 years. Since all wild animal markets have been banned and all wild animals kept in the markets have been slaughtered in China, it is well possible that the intermediate and amplifying hosts of SARS-CoV-2, similar to civets in the case of SARS-CoV, could never be found. In support of this, no wild or farmed civets, except the ones captured in one wild animal market in Shenzhen in 2003, have been found to carry SARS-CoV. However, if a reservoir host for SARS-CoV-2 exists just as dromedary in the case of MERS-CoV, it might take a bit longer to identify it. Only time will tell what is right. Concerning the origin of COVID-19 in Wuhan, we don’t think that we have the first-hand data to make any judgement on patient zero or the role of Huanan Seafood Wholesale Market, other wild animal markets, or any other places. For Huanan Seafood Wholesale Market, it remains elusive as to whether many confirmed cases were linked to a particular type of seafood and whether any infected individual was exposed to wild animal. The existence of a human superspreader in the market should also be seriously investigated. In this regard, it is of some interest to see the recent identification of a female seafood vendor at Huanan Market as the possible patient zero whose symptom onset was on December 10, 2019 [10]. However, since most infected people in Wuhan were asymptomatic and undiagnosed [2] and new cases dated back to December 2019 or even earlier were retrospectively identified, only those who have access to the first-hand data can answer whether she would be the bona fide patient zero. It is not surprising that more patients with symptom onsets earlier than December 10 have been discovered or remain to be discovered. Without thorough reassessment and release of the full dataset, analysis on the early cases in Wuhan remains incomplete and too speculative. Due to the tremendous impact of the pandemic on world economy and public health, it is imperative that Chinese and international experts should be given full access to the key original data of all early patients.

The ninth question concerning why SARS-CoV-2 is less pathogenic remains to be addressed by new thoughts and experiments. This is a long-term goal that will keep us busy in years. In this regard, one top priority is to verify whether and why Omicron variant might be less pathogenic. In addition to the comparative analysis of different viruses of SARS-CoV-2 and SARS-CoV as suggested earlier, comparison of the different hosts might also be instructive. SARS-CoV-2 is non-pathogenic in bats. Comparing bats and humans for interferon antagonism and inflammasome activation capability of SARS-CoV-2 might reveal how bats evade immunopathogenic outcome of SARS-CoV-2 infection. This might enlighten us about what critical pathways and mechanisms should be targeted to relieve SARS-CoV-2 pathogenesis in humans. New knowledge about SARS-CoV-2 pathogenesis particularly in terms of interferon antagonism and inflammasome activation will lay the foundation for rational design and development of vaccines and antivirals against SARS-CoV-2. For example, recombinant SARS-CoV-2 lacking some particular interferon antagonists or inflammasome activators are excellent candidates of live attenuation vaccines. Indeed, NSP16-deficient SARS-CoV-2 is attenuated in vitro and in animals (our unpublished data). NSP16 is a 2’-O-methyltransferase and an interferon antagonist. Further optimization of this candidate strain might result in a live attenuated vaccine against SARS-CoV-2.

SARS-CoV-2 was initially isolated in Wuhan. China should be commended for effectively eliminating SARS-CoV-2 from the communities, keeping them SARS-CoV-2-free for almost two years, producing the highest quantities of vaccines in the world and vaccinating billions of people. These great achievements are shadowed by errors, misjudgments, incompetencies and other weaknesses, particularly seen in the initial phase of the response to the outbreak in Wuhan. Malfunctioning of the National Communicable Disease Reporting System, time lag in recognizing the high transmissibility of SARS-CoV-2 and lack of communication between different groups of healthcare professionals are just a few examples. China was not alone in making some of the mistakes, which were repeated later elsewhere. Chinese inactivated vaccines are safe and effective, particularly after three or more injections. China has provided effective vaccines to Chinese people and many others who have no access to any other SARS-CoV-2 vaccines. In this sense, Chinese inactivated vaccines are saving lives. However, as discussed above, the neutralizing antibody titers elicited by these vaccines are not sufficiently high. Booster injections are required, and alternative strategies to improve their performance and to develop next-generation vaccines should be actively explored. Another open question concerns whether the zero-tolerance policy in the control of COVID-19 is sustainable. The benefits and harms of this policy versus that of “living with SARS-CoV-2” should be carefully considered and openly debated. The tendency of SARS-CoV-2 to become endemic and the low mortality of breakthrough SARS-CoV-2 infection in vaccinees should be taken into full account. Some of the concepts and practices implemented by different countries and economies have their own merits and should not be blindly criticized. For example, Israel and Singapore are taking the lead internationally in the vaccination campaign and in the execution of the “living with SARS-CoV-2” policy. Their hard-learned lessons should be treasured by others. Even if the same policies will not be adopted today, other countries and economies might encounter the same dilemmas and challenges in future.

Above we have revisited the top nine important questions and related issues about SARS-CoV-2 and COVID-19. This pandemic has lasted two years and costed millions of lives. In our opinion, the beginning of the end of the pandemic is already in sight. As in the history, pandemics caused by the other four coronaviruses would be over when the virus became highly adapted in humans and when herd immunity developed in human populations was sufficiently strong. Whereas evidence-based mitigation measures have reduced the damage caused by the pandemic, research efforts have already made a difference in multiple fronts of our battle against SARS-CoV-2 and COVID-19. Importantly, the vaccination schemes have already accelerated the end of the pandemic. Development of next-generation vaccines and innovative use of existing vaccines in combination might ultimately lead to eradication of SARS-CoV-2. We therefore remain guardedly optimistic.

## Data Availability

Not applicable.
